# Gonotrophic discordance in *Culex quinquefasciatus* (Diptera: Culicidae) in the city of São Paulo, Brazil

**DOI:** 10.1590/0037-8682-0277-2019

**Published:** 2019-12-20

**Authors:** Sabrina Ribeiro Santana, Pâmela dos Santos Andrade, Paulo Roberto Urbinatti, Rosa Maria Marques de Sá Almeida, Tamara Nunes Lima-Camara

**Affiliations:** 1 Universidade de São Paulo, Faculdade de Saúde Pública, Laboratório de Entomologia em Saúde Pública, São Paulo, SP, Brasil.; 2 Universidade de São Paulo, Faculdade de Saúde Pública, Departamento de Epidemiologia, São Paulo, SP, Brasil.

**Keywords:** Vector, Culex, Parity, Ovaries

## Abstract

**INTRODUCTION:**

This study aimed to assess the occurrence of gonotrophic discordance in females of *Culex quinquefasciatus* in São Paulo, Brazil.

**METHODS:**

Resting females were collected monthly for 8 months. Females of *Cx. quinquefasciatus* were identified, and their midgut and ovaries were dissected.

**RESULTS:**

Two hundred females were dissected, out of which, 27.5% were nulliparous and 57% were parous. Most females had no blood in the midgut, but gonotrophic discordance was found in 21% females.

**CONCLUSIONS:**

Females of *Cx. quinquefasciatus* showed a high parity rate and gonotrophic discordance, which could favor the vector capacity of this species.

*Culex quinquefasciatus* is widely distributed throughout the American continent, particularly in the southern United States, northern Argentina, and throughout Brazil^1^ ^,^ ^2^. The development of the larvae and pupae of *Cx. quinquefasciatus* occurs mainly in bodies of water with large amounts of organic matter, such as polluted rivers and abandoned wells^1^ ^,^ ^2^. The adults of this species are closely associated with humans and are frequently found inside residences in urban and suburban areas^1^ ^,^ ^2^.

In the United States, *Cx. quinquefasciatus* is involved in the dynamics of transmission of the West Nile Virus (WNV) and Saint Louis Encephalitis Virus (SLEV)^3^. Several studies have elucidated important aspects of biology and ecology of this mosquito species in the US^4^ ^,^ ^5^. 

This species also has epidemiological importance in Brazil because it is considered to be the main vector of the etiological agent of lymphatic filariasis (*Wuchereria bancrofti*) and dirofilariasis (*Dirofilaria immitis*)^2^ ^,^ ^6^. Recently, the WNV was isolated for the first time in Brazil from a horse with neurologic disease, and *Cx. quinquefasciatus* could be an important vector of this virus as well^7^.

The digestion of the ingested blood meal stimulates the development of the ovarioles, which occurs through several stages^8^ ^,^ ^9^. The coiled ends of the tracheoles, which supply the ovaries with oxygen, unfurl during the maturation of eggs as the ovaries increase in size and do not recoil after oviposition. Thus, the observation of the ends of the tracheoles i.e., tightly curled when the female mosquito has never laid eggs (nulliparous) and distended as the female becomes gravid for the first time, can be used to determine parity^10^. The proportion of female mosquitoes that are parous provides an estimate of survival in the population^11^.

Gonotrophic discordance occurs when a female mosquito feeds more than once within an egg-laying cycle^11^. This phenomenon is of epidemiological importance because it increases the contact of mosquito with the vertebrate host and, consequently, the chances of the vector becoming infected and/or transmitting a pathogen that it harbors^11^. 

Owing to the medical importance of *Cx. quinquefasciatus*, the hypothesis of our study was that females of this species show a high parity rate and gonotrophic discordance. Thus, the objective of the current study was to evaluate parity and the presence of gonotrophic discordance in females of *Cx. quinquefasciatus* in an urban area in the city of São Paulo, SP, Brazil. 

Resting *Cx. quinquefasciatus* were collected in the Parque Esportes para Todos, at the University campus of the University of São Paulo (23°33’34.4”S; 46°44’12.8”W). Located in an area containing a remnant of Atlantic Forest, the park is forested, but frequently visited by people, mainly along a trail of about 1 km. 

The monthly collections were taken along the trail in the park during eight-months period (from August 2016 to March 2017), using manual aspirators connected to a 12-volt battery. Mosquitoes were mostly collected from vegetation. Although all collectors with manual aspirators wore long clothing to avoid mosquito bites, yet they attracted some specimens of mosquitoes that were promptly aspirated. The time of aspiration was one and a half hour in the morning per collection day. Collections were taken to the Laboratory of Entomology in Public Health, School of Public Health, University of São Paulo, where the mosquitoes were anaesthetized on ice, and identified and separated by species and sex. The identification of the species was done with the help of the keys in Consoli and Lourenço-de-Oliveira^2^ and Forattini^1^.

The females of *Cx. quinquefasciatus* were placed on a glass slide with a drop of 0.9% NaCl solution for dissection. Their midgut and ovaries were removed under a stereoscopic microscope using the techniques proposed by Consoli and Lourenço-de-Oliveira^2^. Initially, the presence or absence of blood and its coloration (red or brown) were verified and classified as proposed by Lima-Camara et al.^11^. Then, parity (distension of the tracheoles) and the stage of development of the ovaries was determined under an optical microscope (100× magnification) in accordance with Christophers^9^ and Mer’s^8^ classification. The stages of development of the ovaries were initial (N, I, and I-II); intermediate (II); and final (III, IV, and V).

Females of *Cx. quinquefasciatus* with red blood in their midguts and ovaries in the final stages of development were considered to be in gonotrophic discordance, because the recent blood meal could be associated with the initial or intermediate stages of ovarian maturation^11^. Gonotrophic discordance was considered in females of *Cx. quinquefasciatus* with brown blood in midgut and ovaries in the initial and intermediate stages of development as well, because to complete the maturation of the eggs, at least one further blood meal would be necessary. Females with red or brown blood and ovaries in the last stage of development (V) were also considered to be in gonotrophic discordance, because in this stage the female is considered gravid and presents completely digested blood^11^. Females with no blood in their midguts and ovaries in different stages of N, I, I-II, and V were also classified as being in gonotrophic discordance.

Four hundred and ninety specimens of *Cx. quinquefasciatus* were collected, out of which 203 were females and 287 males. Most of the females (173/203; 85.22%) and males (239/287; 83.27%) were collected in December and January (late spring and summer, respectively). Of the 203 females, 200 (98%) had their midgut content, parity and ovarian stage of maturation determined. 

Overall, 57% (114/200) of the females were parous and 27.5% (55/200) were nulliparous (Figure 1). The parity of 31 females of *Cx. quinquefasciatus* (15.5%) could not be determined because the ovaries were in advanced stages of maturation (III-V), precluding the use of the tracheole method to determine parity. 

**FIGURE 1:**
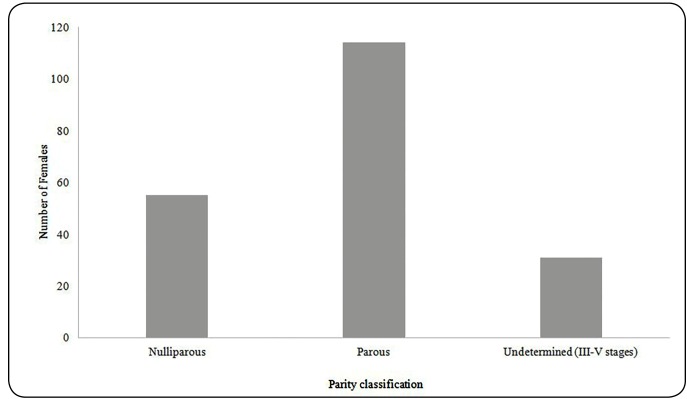
Classification of parity in females of *Cx. quinquefasciatus* collected in a forested area with human circulation in the city of São Paulo, SP, Brazil.

Of the 200 females analyzed, 70.5% (141/200) had no blood in their midgut, while 29.5% (59/200) had blood in their midgut, 28.81% (17/59) of them having red blood and 71.19% (42/59) having brown blood (Table 1). Among the females that did not present blood in their midgut, the ovaries of 89.36% (126/141) were in the initial stages of development (N, I, and I-II), whereas, 10.64% (15/141) presented ovaries in the last stage of development (V) and were ready to oviposit. Of the females with red blood in their midgut, 64.71% (11/17) had ovaries in the initial and intermediate stages of development. Nevertheless, 35.29% (6/17) had ovaries in stages III and IV, indicating gonotrophic discordance. Although 14.29% (6/42) of the females with brown blood in their midguts had ovaries in stages III and IV of development, 76.19% (32/42) had ovaries in the initial and intermediate stages, and 9.52% (4/42) had ovaries in stage V, indicating gonotrophic discordance (Table 1). 

**TABLE 1:** Ovarian stages and presence of blood in the midgut of females of *Cx. quinquefasciatus.*

	N	I	I-II	II	III	IV	V	Total
No blood	50 (25)	73 (36.5)	3 (1.5)	-	-	-	15 (7.5)	**141 (70.5)**
Red	-	7 (3.5)	2 (1)	2 (1)	5 (2.5)	1 (0.5)	-	**17 (8.5)**
Brown	-	18 (9)	13 (6.5)	1 (0.5)	2 (1)	4 (2)	4 (2)	**42 (21)**
**Total**	**50 (25)**	**98 (49)**	**18 (9)**	**3 (1.5)**	**7 (3.5)**	**5 (2.5)**	**19 (9.5)**	**200**

Relation between stage of maturation of the ovaries and the color of the blood found in the midgut of females of *Cx. quinquefasciatus* collected in a forested area, but with human circulation, in the city of São Paulo, SP, Brazil. Numbers in parenthesis represent percentage. **N:** Follicle with eight undifferentiated cells; **I:** The oocyte is clearly visible in the distal portion of the follicle; **I-II:** Yolk granules can be seen around the nucleus of the oocyte; **II:** Numerous yolk granules can be seen and the oocyte occupies up to 50% of the length of the follicle; **III:** The oocyte occupies 50% to 75% of the follicle length; **IV:** The oocyte occupies 90% of the follicle length; **V:** The oocyte reaches its full length and the female is considered gravid.

Based on the criteria used in this study, which related the midgut content and the stages of the development of the ovaries, it was observed that 21% of the females (42/200) of *Cx. quinquefasciatus* were in gonotrophic discordance.

In the current study, resting males and females of *Cx. quinquefasciatus* were collected in a wooded area that is frequented visited by the people in the city of São Paulo. Despite the fact that the majority of females collected had no blood in the midgut, almost 30% of them had had at least one blood meal. We further observed that at least 57% of the females of *Cx. quinquefasciatus* were classified as parous and that 21% were in gonotrophic discordance, thus, validating our hypothesis. 

The presence of 57% of parous females suggests the longevity for this population of *Cx. quinquefasciatus* ^11^. Lower parity rates of *Cx. quinquefasciatus* females have been reported in studies conducted in the United States. Reisen et al^5^ have reported the collection of host-seeking *Cx. quinquefasciatus* females in the Los Angeles Basin of California using Centers for Disease Control and Prevention light traps. A parity rate of 39.6% and a daily survival rate of 0.838 were reported. In Orange and Los Angeles Counties, California, the parity rates of host-seeking and resting *Cx. quinquefasciatus* females were 46.4% and 36.1%, respectively^4^. 

In Brazil, David et al.^12^ undertook the collections of *Cx. quinquefasciatus* in three distinct environments (a middle-class area, suburban area, and slum) in the city of Rio de Janeiro. Parity rates varied greatly according to the study area, ranging from 64% to 93.75% in the middle-class area, 36.4% to 78.5% in the suburban area, and 40% to 73.3% in the slum. The daily rate of survival calculated for *Cx. quinquefasciatus* in the middle class area was the greatest, confirming that this area was most favorable to longevity of this species^12^.

 In the current study, 63% of *Cx. quinquefasciatus* females had no blood in the midgut and had ovaries that were in the early stages of maturation whereas 27.5% were blood-fed and 9.5% were gravid. Similar results were reported by Reisen et al.^4^, who observed 49% of *Cx. quinquefasciatus* females without blood and ovaries in early stages of maturation, and 23% and 28% of females with blood-fed/ovaries up to stage IV and gravid status, respectively.

All the females of *Cx. quinquefasciatus* without blood in their midguts were considered to be in gonotrophic concordance because they presented ovaries in the initial stages or in stage V of development. Females with brown and red blood in the midgut and ovaries in the early and late stages of development, respectively, were also considered to be in gonotrophic discordance. With a small volume of blood in the advanced stage of digestion and ovaries in early stages of maturation, the females of *Cx. quinquefasciatus* would probably need at least one more blood meal to become gravid, which also indicates gonotrophic discordance. A large volume of red blood and ovaries in late stages of maturation suggest that females of *Cx. quinquefasciatus* had fed on blood recently to complete the development of the eggs.

Taking several blood meals within the same egg-laying cycle is of great epidemiological importance because it suggests that increased contact of the vector with the host would augment the chances of acquiring and transmitting a pathogen^11^.

Charlwood^13^, while analyzing the host-seeking behavior of females of *Cx. quinquefasciatus* in Manaus, Amazonas, Brazil, observed that the majority of females, which were attracted by human bait, were not blood fed. However, 5% of the females of *Cx. quinquefasciatus* attracted by human bait were partially blood fed, suggesting gonotrophic discordance^13^.

The investigation of parity and the presence of gonotrophic discordance in females of *Cx. quinquefasciatus* would help to explain the transmission of pathogens that this species can harbor. *Cx. quinquefasciatus* is associated mainly with the discomfort caused by its bites and with the transmission of microfilariae in specific localities in Brazil^2^. However, this mosquito is also involved in the dynamics of transmission of the WNV and SLEV in the United States^3^, and the circulation of these viruses in human beings has already been reported in Brazil^14^ ^,^ ^15^. Thus, the importance of *Cx. quinquefasciatus* for public health should not be disregarded, since it can be a vector of various human pathogens, as well as the cause of discomfort because of its night time biting behavior. 

Further studies on the biology and reproductive characteristics of *Cx. quinquefasciatus*, such as parity, multiple blood meals, and gonotrophic state, are of extreme importance to understand this mosquito better and to apply more adequate strategies of vigilance and control.
